# Impact of short and long-term application of low-level laser therapy on mandibular alveolar process of osteoporotic rats – a Histological and Molecular Study

**DOI:** 10.1007/s10103-024-04246-1

**Published:** 2025-01-03

**Authors:** Mai O. El-Halwagy, Enas M. Hegazy, Hany K. Shalaby, Elham F. Mahmoud

**Affiliations:** 1 Oral Biology Department, Faculty of Dentistry, Suez University, P.O.Box:43221, Suez, Egypt; 2 Oral Biology Department, Faculty of Dentistry, Suez Canal University, P.O.Box:41523, Ismailia, Egypt; 3 Oral Medicine and Periodontology Department, Faculty of Dentistry, Suez University, P.O.Box:43221, Suez, Egypt

**Keywords:** Osteoporosis, Dexamethasone, Low-level laser therapy, Alveolar bone, Real-time PCR

## Abstract

This study aims to investigate and compare the effects of short and long-term application of low-level laser therapy on the mandibular alveolar process of osteoporotic rats. Forty adult male albino rats were included in this study. After animal grouping, the experimental group received dexamethasone (0.1 mg/kg b.wt./day for 60 days) for the induction of osteoporosis, then the rats were treated using LLLT (830 nm, 100 mW, at 60 J/cm^2^). The lower jaw specimens were collected and processed for histological, molecular, and histomorphometric assessments. The osteoporotic group exhibited alveolar bone resorption, accompanied by significantly upregulated RANKL and downregulated OPG mRNA expression. The short-term application of laser group showed alveolar bone partial improvement with slightly downregulated RANKL and slightly upregulated OPG levels. The long-term application of laser group showed dramatic positive changes in the alveolar bone, with markedly downregulated RANKL and upregulated OPG levels. LLLT shows potential as a low-risk and impactful local management for osteoporosis, with long-term laser application demonstrably improving bone quality, quantity, and organization compared to short-term application.

**Graphical abstract:**

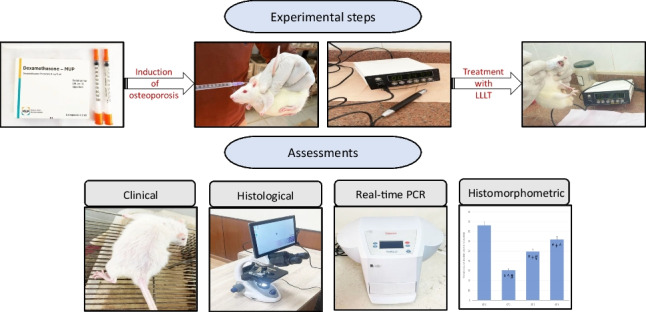

## Introduction

Osteoporosis is a systemic skeletal disease associated with low bone mineral density (BMD) and deterioration of bone microarchitecture that results in increased bone fragility and susceptibility to fractures [1].

In the last few decades, osteoporosis has gained increased attention on a global scale [2], as it affects over 200 million people worldwide, and that number is rapidly growing [3]. With the rapid rise of aging populations in many countries, osteoporosis threatens to become a global health concern, potentially impacting the quality of life for millions [4].

The etiology of osteoporosis determines its classification as primary and secondary. Primary osteoporosis can be further categorized into two subtypes: type I, associated with menopause, and type II, associated with aging [5]. In secondary osteoporosis, glucocorticoids are considered the most prevalent medications associated with drug-induced osteoporosis [6].

Reducing bone loss and maintaining bone density are the goals of osteoporosis treatment. A combination of anabolic and antiresorptive drugs is currently used to treat osteoporosis. Despite advancements, many patients refuse to take antiresorptive drugs due to their adverse effects and the absence of perfect proof that their long-term efficacy will continue [7].

Numerous physical therapy techniques, including whole-body vibration, pulsed electromagnetic field, exercise, and low-level laser therapy (LLLT), have demonstrated efficacious outcomes in the management of osteoporosis [8].

Since the 1970s, with the increased availability of laser technology, research has explored its potential as a treatment for various connective tissues. LLLT is laser light absorption at the electronic level, without heat generation [9], sometimes referred to as photobiomodulation (PBM), which is a safe, non-invasive modality possessing demonstrably robust anti-inflammatory effects [10].

Low-power laser irradiation exhibits unique properties, demonstrably influencing cellular metabolism (bio-stimulation), alleviating pain (analgesia), enhancing wound healing (regeneration or repair), and modulating edema and inflammation. Thus, it has been used in dentistry and medicine as a therapeutic technique for a variety of conditions [11].

Applications for LLLT have been utilized for new bone reconstruction applications in the tibia, femur, humerus, radius, and mandible, among other body parts [12]. Recently, LLLT has emerged as a treatment for osteoporosis [13]. Low-level laser irradiation exhibits biostimulant effects, expanding the bone organic matrix and increasing the osteoblast mitotic index, resulting in an increased number of differentiated osteoblasts, enhanced activity, and accelerated bone formation [14].

The receptor activator of nuclear factor kappa B ligand (RANKL) and its receptor RANK, proteins belonging to the tumor necrosis factor family, regulate bone remodeling by promoting osteoclast development and activation. The balance between RANKL and its decoy receptor, osteoprotegerin (OPG), maintains bone homeostasis, with OPG binding to RANKL, thus limiting the interaction between RANKL and RANK and inhibiting osteoclast differentiation [15].

The RANKL/OPG ratio serves as a pivotal determinant of bone resorption; upregulation of RANKL and downregulation of OPG, both independently, induce bone loss [16]. The regulation of the RANKL/RANK/OPG pathway is influenced by numerous endogenous variables, including mesenchymal transcription factors, hormones like vitamin D, estrogen, and glucocorticoids, as well as certain cytokines [17].

The application of LLLT in the biostimulation of alveolar bone regeneration has been increasing over time, and several studies have demonstrated its positive influence on bone tissue healing [18]. However, further investigations are needed to definitively establish the therapeutic efficacy and optimal treatment parameters of LLLT to be used in clinical therapies.

The present study aims to investigate and compare the effects of short and long-term application of low-level laser therapy on the mandibular alveolar process of osteoporotic rats.

## Materials and methods

### Study procedures

The present research was an experimental study conducted after the approval of the Research Ethics Committee of the Faculty of Dentistry, Suez Canal University (approval number: 403/2021). Following the ethical guidelines of animal care.

### Sample size calculation

Using the G-Power statistical power analysis program (version 3.1.9.7) for sample size determination [19], A sample size of 40 (10 per group) was sufficient to detect a large effect size ranging from 0.55 to 0.57, with an actual power (1-β error) of 0.8 (80%) and a significance level (α error) of 0.05 (5%) for the two-sided hypothesis test.

### Study settings

Forty adult male albino rats with an average body weight of 160-180gm were included in this study. They were labeled numerically and kept 5/cage. They were fed an adequate natural diet consisting of fresh vegetables and dried bread and given drinking water ad libitum, and they were kept under proper conditions of controlled temperature at 27–30°C.

### Induction of osteoporosis

Osteoporosis was induced using dexamethasone 0.1 mg/kg b.wt./day diluted in saline subcutaneously in rats for 60 days [20].

### Osteoporosis confirmation

After 60 days of induction, lower jaw specimens were dissected, decalcified, and processed for hematoxylin and eosin (H&E) staining to confirm osteoporosis induction through histological examination.

### Animal grouping and treatment protocol

**Group 1 (control group)**: consisted of 10 rats who served as negative controls. The rats received a normal diet and distilled water. Half of the rats were euthanized with the short-term application of laser group (67th day), while the other half were euthanized with the long-term application of laser group (85th day).

**Experimental group**: consisted of 30 rats who were administered dexamethasone subcutaneously at a dose of 0.1 mg/kg b.wt./day diluted in saline for 60 days for the induction of osteoporosis. After the induction of osteoporosis, the experimental group was subdivided into three main groups: group 2 (osteoporotic), group 3 (short-term LLLT), and group 4 (long-term LLLT), as follows:

- **Group 2 (osteoporotic group)**: consisted of 10 osteoporotic rats; they served as positive controls and were left without any treatment. Half of the rats were euthanized with the short-term application of laser group (67th day), while the other half were euthanized with the long-term application of laser group (85th day).

- **Group 3 (short-term application of laser group):** consisted of 10 osteoporotic rats that were exposed to LLLT sessions [830 nm, continuous wave, 0.028 cm^2^ beam diameter, 100 mW, at 60 J/cm^2^ with an irradiation time of 34 s] [21] on the lower jaw, at the molar area, every 48 h for 7 days [22].
- **Group 4 (long-term application of laser group):** consisted of 10 osteoporotic rats, which were treated the same way as the short-term application of laser group but for 25 days [22].

### Euthanization method

Rats were euthanized after finishing the treatment protocol for each group by overdose inhalation of ether.

## Methods of evaluation

The experiment lasted for 67 days for the short-term application of laser group, then the rats were euthanized, and 85 days for the long-term application of laser group. Each rat's lower jaw was dissected and separated into two halves, then fixed in 10% neutral buffered formalin for 72 h. Subsequently, decalcification was performed in a 10% EDTA solution at pH 7.4 with daily solution changes for 2–3 weeks.

After complete decalcification, the specimens of the molar region were processed and embedded in paraffin. Five-micron-thick sections were cut and stained with hematoxylin and eosin (H&E) for histological examination to detect any structural changes in the mandibular alveolar process. The slides were examined under a light microscope with a built-in digital camera (OPTIKA digital binocular brightfield microscope).

Representative lower jaw specimens from the different groups were collected, washed in phosphate-buffered saline, and stored in liquid nitrogen at −80°C for real-time PCR assessment of RANKL and OPG mRNA expression.

### Preparation for histological evaluation

To prepare the decalcified samples for microscopic examination, they were dehydrated through graded alcohols, cleared with xylene, and embedded in paraffin wax. Five-micron sections were prepared using a rotary microtome for analysis of potential structural variations of the mandibular alveolar process across samples of different groups. These sections were then stained with a standard H&E stain for further analysis under a light microscope.

### Preparation for real-time PCR evaluation

**RNA extraction:** pure RNA was extracted using a total RNA purification kit following the manufacturer protocol (Thermo Scientific, Fermentas, #K0731). RNA isolation involved homogenizing the samples in a lysis buffer containing guanidine thiocyanate, a chaotropic salt that disrupts cellular structures and inactivates RNases, enzymes that degrade RNA. The lysate was then mixed with ethanol to facilitate RNA binding to a silica membrane within a purification column. The remaining cellular components passed through the column during centrifugation. Subsequent washes with specific buffers eliminated impurities. Finally, under low-ionic conditions, pure RNA was eluted from the column using nuclease-free water for further analysis.

**Complementary DNA (cDNA) synthesis:** this technique utilized reverse transcription kits (Thermo Scientific™, Fermentas, #EP0451) to make a DNA (cDNA) copy of the RNA. The kits specifically employed RevertAid™ H Minus Reverse Transcriptase enzyme for this process.

**Real-time PCR:** to determine the mRNA expression levels, real-time PCR with SYBR green was employed. β-actin was used as an internal reference for normalization. The analysis utilized commercially available Maxima SYBR Green/ROX qPCR Master Mix (Thermo Scientific, USA, #K0221) and gene-specific primers to amplify the previously isolated cDNA. Primer3, a web-based tool, was used to design specific primers. These primers were created using published rat gene sequences as a reference. To ensure the primers wouldn't amplify unintended sequences, we verified their specificity against a vast database of known sequences with the Basic Local Alignment Search Tool (BLAST) [23]. The final reaction mixture was placed in a PikoReal™ 24 real-time PCR system (Thermo Scientific™, USA).

The relative gene expression or fold change in the target genes was estimated using the housekeeping gene β-actin as a reference point. Accordingly, the 2^−∆∆Ct^ method was employed to normalize the quantities critical threshold (Ct) of the target genes with the quantities Ct of the housekeeping gene [24] as follows:

The control group was used as a calibrator, while other groups were represented as test groups in both target and reference genes. The Ct cycle numbers of target genes were normalized to those of reference (ref.) genes in both the test groups and the control group by using the following equations:

$$\Delta \mathrm{Ct}\left(\mathrm{test}\right)=\mathrm{Ct}\left(\text{target in test groups}\right)-\mathrm{Ct}\left(\mathrm{ref}.\text{ in test groups}\right)$$

$$\Delta \mathrm{Ct}\left(\mathrm{calibrator}\right)=\mathrm{Ct}\left(\text{target in control}\right)-\mathrm{Ct}\left(\mathrm{ref}.\text{ in control}\right)$$

The ∆Ct of the test genes was normalized to the ∆Ct of the calibrator: ∆∆Ct = ∆Ct (test)—∆Ct (calibrator).

The fold change of relative gene expression was calculated: fold change = (2^−∆∆Ct^).

### Preparation for histomorphometric evaluation

A scoring system was implemented and adjusted to evaluate the status of the alveolar bone [25];

- Score 1-normal: regular borders with no signs of osteoclastic activity.
- Score 2-mild: regular borders with minimal signs of osteoclastic activity.
- Score 3-moderate: irregular borders with occasional osteoclastic activity.
- Score 4-severe: irregular borders, numerous osteoclasts and sequestration.

Quantitative scoring was evaluated for trabecular bone volume in H&E-stained sections. The image analysis system Leica QWin DW3000 (Leica Imaging Systems Ltd., England) was used to analyze the scores based on the cross-sectional area of each sample. This analysis involved examining six representative fields for each section among all groups under a light microscope at 200X magnification. Histomorphometry of the mandibular alveolar process was performed specifically in the interradicular septum of the second molar to measure trabecular bone volume in osteoporotic rats compared to controls.

## Statistical analysis

Numerical data were described as means ± S.E. Statistical significance was assessed using one-way ANOVA (analysis of variance) followed by Tukey–Kramer post-hoc (Duncan's multiple range test, DMRT) tests for intergroup relationships, performed with SPSS version 26.0. A p-value < 0.05 indicated statistical significance.

## Results

### Clinical observations

#### Mortality rate

All of the rats were alive throughout the experiment.

#### Hair loss (Fig. 1a, b, c, d)

**Fig. 1:**
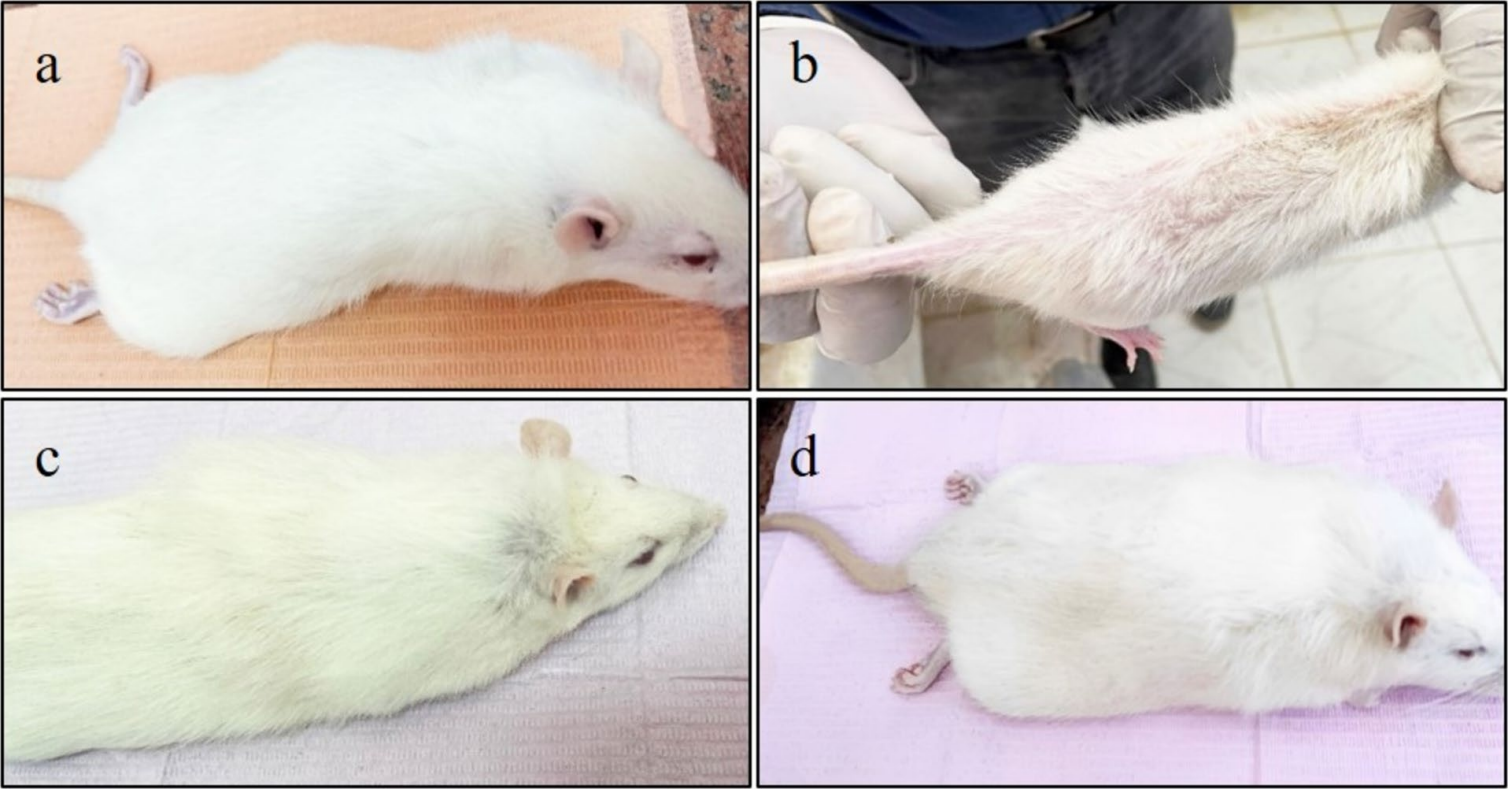
**a**, **b**, **c** & **d**: Photographs illustrate the hair distribution of different groups of rats. **a**: Control group with normal hair distribution. **b**: Osteoporotic group with marked and severe hair loss in conjunction with dexamethasone injection. **c**: Short-term application of laser group with partial regaining of hair. **d**: Long-term application of laser group with marked improvement in hair distribution

There was significant and severe hair loss in conjunction with dexamethasone injection for the induction of osteoporosis. With the stoppage of dexamethasone and the beginning of the treatment with LLLT, the rats gradually regained their normal hair distribution compared with the control group.

### Histological results (Fig. 2a, b, c, d)

**Fig. 2:**
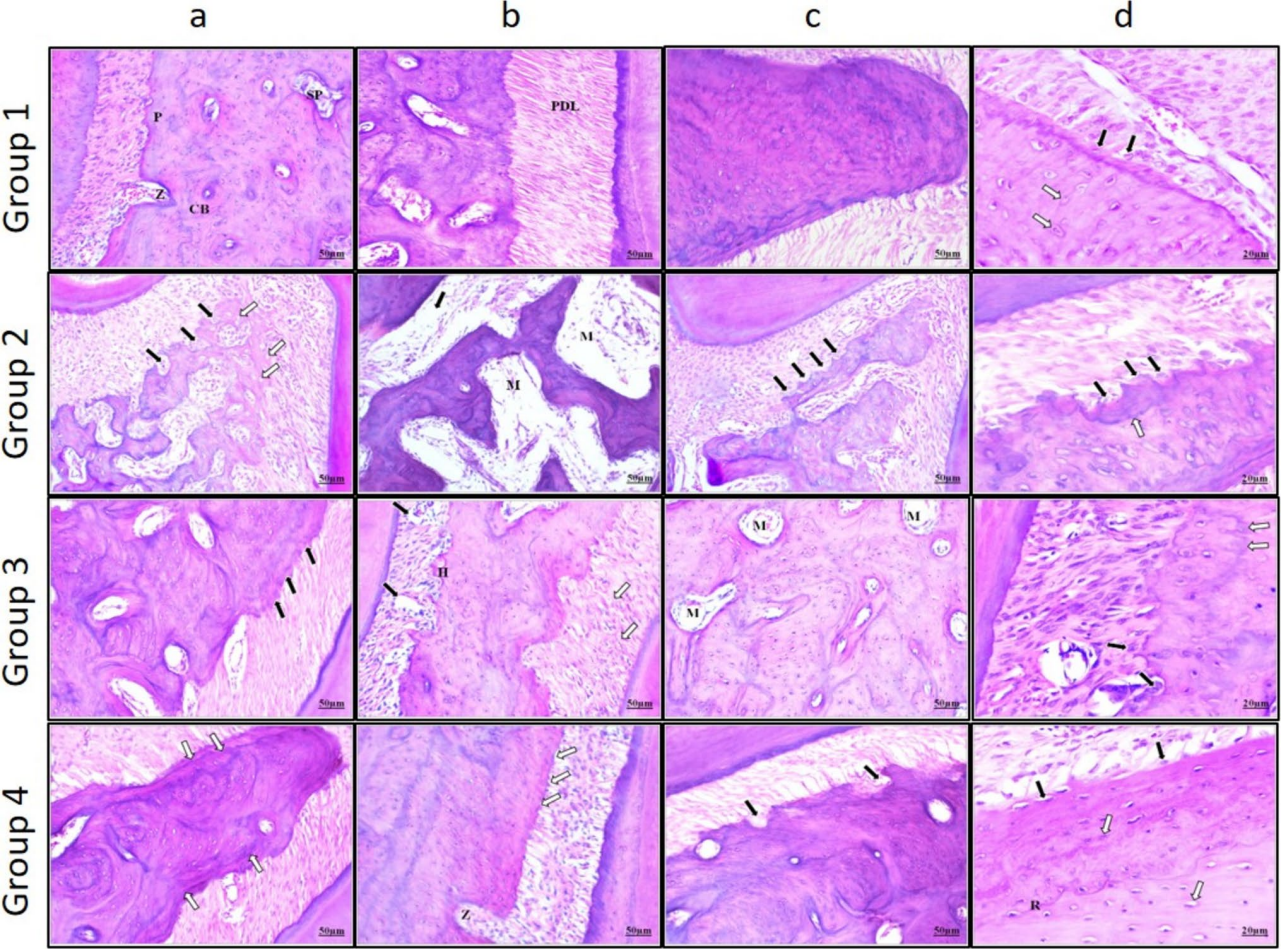
**a**, **b**, **c** & **d**: Photomicrographs of the alveolar bone of different rat groups. Group 1: **a**: Alveolar bone proper (P), including compact bone (CB), supporting alveolar bone composed of spongiosa (SP), and Zuckerkandle and Hershfield canal (Z). **b**: Well-organized PDL fibers (PDL) inserted in the alveolar bone. **c**: Smooth and regular alveolar margins with slightly undulated, well-defined resting lines. **d**: Plump osteoblasts (black arrows) at the alveolar margins and normal osteocytes (white arrows) in their lacunae. Group 2: **a**: Bone resorption with irregular, scalloped alveolar margins, a lot of osteoclasts (black arrows), and empty osteocyte lacunae (white arrows). **b**: Thin, irregular bony trabeculae enclosing wide marrow cavities (M) and detachment of the PDL fibers (arrow). **c**: Irregular resorbed alveolar margins with a lot of osteoclasts Howship’s lacunae (arrows). **d**: Reversal lines (white arrow) denote a high rate of bone resorption and a lot of osteoclasts (black arrows). Group 3: **a**: Partial improvement of alveolar bone with newly formed bone (arrows) and a characteristic mosaic appearance. **b**: Relatively irregular alveolar margins with high osteoclastic activity in their Howship’s lacunae (H), some degenerated areas of PDL fibers (black arrows), and others regained their normal orientation (white arrows). **c**: Thicker bony trabeculae enclosing narrower marrow cavities (M) lined by osteoblasts. **d**: A lot of osteoclasts (black arrows) with multiple resorption pits and reversal lines (white arrows). Group 4: **a**: Marked improvement of alveolar bone with multiple resting lines (arrows) denoting regeneration. **b:** Regular alveolar margins with normal osteoblastic lining (arrows) and Zuckerkandle and Hershfield canal (Z). **c**: Thick bony trabeculae arranged in regular networks and empty Howship’s lacunae denoting decreased osteoclastic activity (arrows). **d**: Plump osteoblasts (black arrows) lining the osteoid tissue and osteocytes (white arrows) appeared with normal size and distribution and the presence of reversal lines denoting new bone formation (R)

#### Group 1 (control group) score 1

The control group rats exhibited the architecture of alveolar bone, as it was formed of alveolar bone proper and supporting alveolar bone. The alveolar bone proper was composed of bundle bone, which was the inner wall of the sockets, containing the openings of Zuckerkandle and Hirschfeld canals, and lamellar bone adjacent to it, where the lamellae were arranged either parallel to each other or in the form of haversian systems. The supporting alveolar bone was composed of spongiosa.

The control group exhibited well-organized, healthy, and continuous periodontal ligament (PDL) fibers inserted into the alveolar bone that showed smooth and regular alveolar margins with thick, dense bony trabeculae enclosing narrow cellular marrow cavities lined by osteoblasts.

The alveolar bone showed a normal rate of bone turnover with evidently well-defined resting lines. Higher magnification revealed a mix of plump and flattened osteoblasts, as well as a normal osteocyte appearance.

#### Group 2 (osteoporotic group) score 4

In the osteoporotic group of rats, bone resorption was the most observed effect, with massive destruction of alveolar bone. This group revealed detachment of the PDL fibers inserted in the alveolar bone that showed eroded, scalloped, and irregularly resorbed alveolar margins, with a lot of multinucleated osteoclasts identified in their Howship’s lacunae. The bony trabeculae were thin and irregular, enclosing wide marrow cavities.

The alveolar bone showed a high rate of bone resorption with many reversal lines. Higher magnification showed cellular degeneration with the absence of osteoblastic lining, while some regions showed flattened osteoblasts, osteocyte lacunae appeared widened, and others were empty. In addition, many osteoclasts with multiple resorption pits were detected.

#### Group 3 (short-term application of laser group) score 3

The short-term application of laser group rats showed partial improvement of alveolar bone architecture, while degeneration in some areas was still detected. This group revealed some degenerated areas within the PDL, fibers while other areas regained their normal orientation. The alveolar bone showed relatively eroded and irregular alveolar margins, with thicker bony trabeculae enclosing narrower marrow cavities, which gradually regained their normal size and shape.

Several reversal lines were observed, along with a lot of resting lines, giving the bone a characteristic mosaic appearance, representing an abnormally high rate of bone turnover. Higher magnification revealed increased osteoblastic activity and a slight increase in the number of osteocytes, while the number of osteoclasts remained high.

#### Group 4 (long-term application of laser group) score 2

The long-term application of laser group rats showed marked improvement and dramatic positive changes in alveolar bone architecture. This group revealed a normal orientation of the PDL fibers inserted in the alveolar bone that showed relatively smooth, slightly undulated, and regular alveolar margins with decreased osteoclastic activity, leaving empty Howship’s lacunae. The bony trabeculae increased in thickness, dense, arranged in regular networks, and enclosed narrower marrow cavities with a well-defined osteoblast lining.

The presence of the reversal lines also decreased, indicating a nearly normal bone turnover rate. To some extent, the alveolar bone of this group regained its normal appearance, with multiple resting lines denoting regeneration and marked bone formation. Higher magnification showed plump osteoblasts lining the newly formed bone, and osteocytes appeared to have normal size and distribution.

### Real-time PCR results

To verify the role of LLLT in osteoporotic bone healing, relative RANKL, and OPG mRNA expressions were detected in the examined bone tissues among all the groups.

#### RANKL real-time PCR results (Fig. 3a, b, c)

**Fig. 3:**
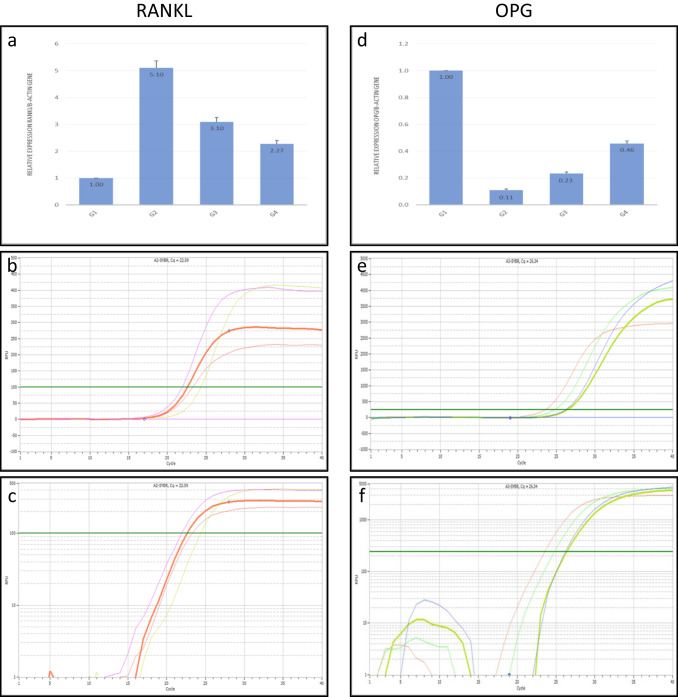
**a**, **b**, **c**: RANKL real-time PCR results. **a**: Graphical presentation of real-time quantitative PCR analysis of the expressions of RANKL showing statistically significant (P ≤ 0.05) upregulation in group 2, followed by group 3, then group 4 compared with group 1 (the control group). **b** & **c**: Linear (**b**) & log (**c**) views of the amplification curves representing the Ct values of RANKL. **d**, **e**, **f**: OPG real-time PCR results. **d**: Graphical presentation of real-time quantitative PCR analysis of the expressions of OPG showing statistically significant (P ≤ 0.05) downregulation in group 2, followed by group 3, then group 4 compared with group 1 (the control group). **e** & **f**: Linear (**e**) & log (**f**) views of the amplification curves representing the Ct values of OPG

The changes in the relative expressions of RANKL between groups were considerable, and the differences in the RANKL expressions were statistically significant **(P < 0.05)** compared with the control group. The osteoporotic group results revealed a marked upregulation **(5.10 ± 0.26)** in the RANKL mRNA level, a key factor known to induce osteoclastogenesis, compared with the control group.

In the short-term application of laser group, the mRNA level of RANKL was slightly downregulated **(3.10 ± 0.17)**, while in the long-term application of laser group, the mRNA level of RANKL was markedly downregulated **(2.27 ± 0.14)** than the osteoporotic group with statistically significant differences.

So, RANKL expressions were significantly upregulated **(P < 0.05)** in group 2, followed by group 3, then group 4, compared with group 1, the control group **(**Table 1**)**.

**Table 1:** Calculations of fold change for the relative expressions of the RANKL gene

Group	Gene average Ct	Delta Ct	Delta delta Ct	Fold change ± SE
Group 1	24.34	−0.05	0.00	1.00 ± 0
Group 2	21.93	−2.40	−2.35	5.10 ± 0.26
Group 3	22.59	−1.68	−1.63	3.10 ± 0.17
Group 4	23.28	−1.23	−1.18	2.27 ± 0.14

#### OPG real-time PCR results (Fig. 3d, e, f)

The differences in the OPG expressions among groups were statistically significant **(P < 0.05)** compared with the control group. The real-time PCR results showed that the mRNA level of OPG in the control group was significantly high as expected, while in osteoporotic rats, the mRNA level of OPG was markedly downregulated **(0.11 ± 0.01)** in comparison with the control group.

In the short-term application of laser group, the mRNA level of OPG was slightly upregulated **(0.23 ± 0.01)**, while in the long-term application of laser group, the mRNA level of OPG was markedly upregulated **(0.46 ± 0.02)** than the osteoporotic group with statistically significant differences.

So, OPG expressions were significantly downregulated **(P < 0.05)** in group 2, followed by group 3, then group 4, compared with group 1, the control group **(**Table 2**)**.

**Table 2:** Calculations of fold change for the relative expressions of the OPG gene

Group	Gene average Ct	Delta Ct	Delta delta Ct	Fold change ± SE
Group 1	23.49	−0.90	0.00	1.00 ± 0
Group 2	26.34	2.31	3.21	0.11 ± 0.01
Group 3	26.17	1.20	2.10	0.23 ± 0.01
Group 4	24.74	0.23	1.13	0.46 ± 0.02

### Histomorphometric results (Fig. 4)

**Fig. 4:**
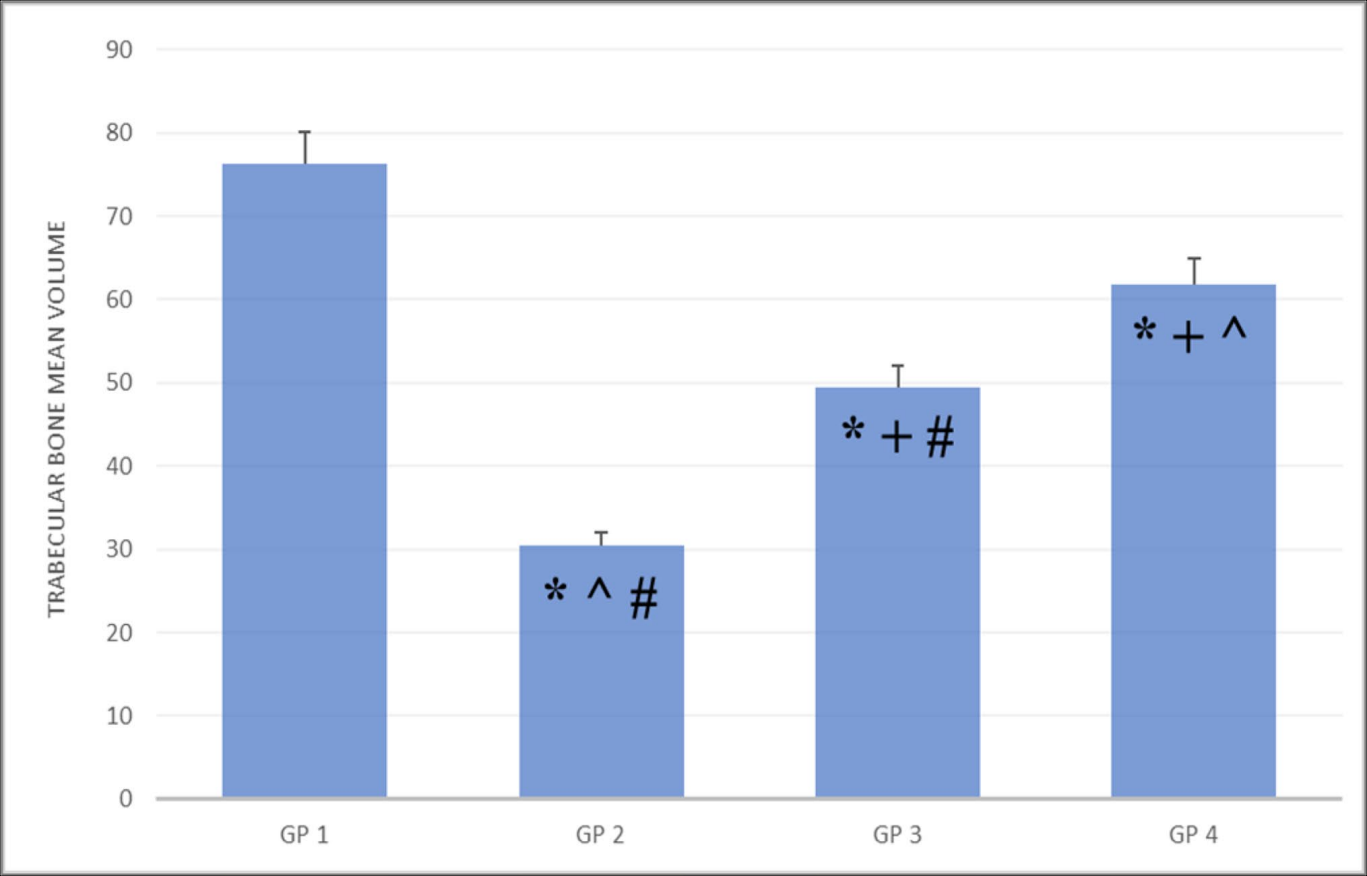
Graphical presentation showing values of trabecular bone volume quantitative scoring expressed as Means ± SE (Area%). The positive mean area% was significantly downregulated in group 2, followed by group 3, then group 4 compared with group 1 (the control group), * significant vs. group 1, + significant vs. group 2, ^ significant vs. group 3, and # significant vs. group 4. Different superscripts (+ , #, ^, *) indicated significant differences at p-value ≤ 0.001

Since histomorphometry is considered the gold standard for quantitative bone analysis in many animal studies, this method was employed to assess the trabecular bone volume of each group.

The highest mean of trabecular bone volume was recorded in the control group **(76.250 ± 0.775)** score 1, followed by the long-term application of laser group **(61.834 ± 3.518)** score 2. The lowest mean of trabecular bone volume was recorded in the osteoporotic group **(30.498 ± 0.985)** score 4, followed by the short-term application of laser group **(49.517 ± 1.410)** score 3.

A statistically significant **(P < 0.05)** difference was revealed between all groups where the positive mean area% was significantly downregulated in group 2, followed by group 3, then group 4 compared with group 1, the control group **(**Table 3, 4**)**.

**Table 3:** One-way ANOVA test of the trabecular bone volume

Group	Mean ± SE
Group 1	76.250 ± 0.775
Group 2	30.498 ± 0.985
Group 3	49.517 ± 1.410
Group 4	61.834 ± 3.518
F level	94.330
P level	0.000

**Table 4:** Tukey HSD (Harmonic Mean Sample Size = 6.000) test of the trabecular bone volume

Group	Subset for alpha = 0.05			
	1	2	3	4
Group 2	30.498			
Group 3		49.517		
Group 4			61.834	
Group 1				76.250
Significance	1.000	1.000	1.000	1.000

## Discussion

Osteoporosis is the most prevalent degenerative bone disease worldwide [26]. It has to do with bone mass loss, which causes bone fragility and raises the risk of fractures, followed by high rates of morbidity and reducing the patient’s quality of life [27].

The progression of osteoporosis can be slowed, stopped, or even reversed in certain cases with the proper management, even though there is no full cure for osteoporosis [28]. Recently, LLLT has emerged as a promising physical therapy that is beneficial for alveolar bone repair because it can cause vascular sprouting, lessen pain and inflammation, and thus speed up the formation of new bone matrix [29].

Clinical results confirmed significant and severe hair loss associated with dexamethasone injection, corroborating reports by other authors [30]. By the beginning of treatment with LLLT, rats had gradually regained their normal hair distribution. The FDA approved LLLT as a safe treatment for hair loss that significantly improves overall hair regrowth, slows down hair loss, and produces thicker-feeling hair [31].

In the present study, the microscopic examination of the control group demonstrated a normal architecture of alveolar bone with well-organized and continuous PDL fibers. Our findings align with another study demonstrating healthy alveolar bone structure in the control group, including healthy continuous PDL fibers, normal thickness of bony trabeculae with smooth margins lined by osteoblasts, and osteocytes appeared at their typical size and distribution [32].

Regarding the osteoporotic group, bone resorption was the most observed effect, with massive destruction of alveolar bone. The alveolar margins appeared eroded, with thin bony trabeculae enclosing wide marrow cavities and numerous multinucleated osteoclasts. It was documented that glucocorticoids increase bone resorption; by reducing OPG, increasing RANKL and reactive oxygen species [33]. Glucocorticoids prevent the differentiation of mesenchymal precursor cells into osteoblasts and have a direct anti-apoptotic effect on mature osteoclasts, increasing their survival and activity [34, 35].

The osteoporotic alveolar bone in the present research showed a high rate of bone resorption; many reversal lines were observed, and some resting lines were spotted. It was suggested that glucocorticoids stimulate high osteoblastic and osteoclastic activities in bone turnover, with the latter activity far exceeding the former [36].

The histological examination of the short-term application of laser group showed partial improvement of alveolar bone architecture, while degeneration in some areas was still detected. Studies have shown that LLLT can significantly increase the expression of genes involved in inflammation and angiogenesis. This promotes faster resolution of inflammation, preventing excessive tissue damage. Additionally, LLLT accelerates bone healing by stimulating the differentiation of pre-osteoblastic cells into mature osteoblasts [37].

Several reversal lines were observed in group 3, besides a lot of resting lines giving the bone a characteristic mosaic appearance, representing an abnormally high rate of bone turnover. It was demonstrated that LLLT can independently accelerate the bone remodeling process with a high rate of bone turnover [38].

In the present study, the long-term application of laser group showed marked improvement and dramatic positive changes in alveolar bone architecture. The enhanced bone healing observed in LLLT groups might be attributed to its ability to stimulate osteoblastic cell activity, including proliferation and maturation, while simultaneously suppressing osteoclast differentiation [39]. LLLT can promote the entry of β-catenin into the nucleus and activate the osteogenic effect of the Wnt signaling pathway to induce bone formation and inhibit bone resorption [40].

According to this study, long-term application of LLLT may have more favorable effects against the osteoporotic mandibular alveolar process of rats than short-term application, as LLLT induced a biomodulatory favorable effect on the bone healing process, which was dependent on irradiation wavelength and time. It was demonstrated that additional variables based on the frequency and timing of the laser irradiation, in addition to the overall laser dosage, influence its effectiveness [41].

As an alternative method to define osteoclastic activity, it was possible to evaluate RANKL, an osteoclast differentiation factor that is necessary for osteoclastogenesis, and OPG, a novel inhibitor of osteoclastogenesis that prevents osteoclast differentiation and maturation [42]. So, the mRNA expression levels of RANKL and OPG were analyzed in bone tissues using real-time PCR.

In the current study, real-time PCR revealed a marked upregulation of RANKL mRNA and downregulation of OPG mRNA in the osteoporotic group compared with the control group. It was suggested that dexamethasone significantly upregulated RANKL and downregulated OPG mRNA transcript levels [43].

However, LLLT groups showed significant downregulation of RANKL and upregulation of OPG mRNA expression levels compared with the osteoporotic group. The presence of PBM significantly decreased osteoclastogenesis by upregulating anti-osteoclastogenic OPG expression and downregulating pro-osteoclastogenic RANKL expression, indicating that PBM exerts an anti-osteoclastogenic effect by modulating the RANKL/OPG pathway [44].

As the gold standard for quantitative bone architecture imaging, histomorphometry was employed to determine the trabecular bone volume of each group in this study. The highest mean of trabecular bone volume was recorded in the control group, while the lowest mean of trabecular bone volume was recorded in the osteoporotic group. It was observed that glucocorticoids could change the properties and composition of trabecular bone, in addition to bone structure deterioration [45].

The present study results showed that the short-term application of laser group recorded a slight increase in the mean of trabecular bone volume, while the long-term application of laser group recorded a marked increase in the mean of trabecular bone volume compared with the osteoporotic group. Some authors observed that, compared to control rats, the volume of tissue repair, the total volume of trabeculae, and the area of collagen increased after 7 days of LLLT [39]. On the other hand, others showed that following 4 weeks of therapy, LLLT increased trabecular bone volume [46].

The applied laser energy is hypothesized to have stimulated cellular processes involved in bone formation, leading to an increase in total collagen content and a greater volume of newly formed bone [39].

To sum up, the data from our investigation revealed that the use of LLLT has a marked healing impact on osteoporotic alveolar bone. In addition, the long-term application of LLLT may have more favorable therapeutic effects than the short-term application. On the other hand, the precise role of laser irradiation in bone remodeling is still not fully understood. So, further investigations are still needed.

## Recommendations

A recovery period is recommended to be added following the low-level laser therapy protocol to determine whether the laser osteogenic effect is temporary or permanent without reversal to the osteoporotic state.

Further research is recommended using the TUNEL technique for the apoptotic analysis of osteoclasts as an assessment method for determining the degree of bone resorption.

The standards set in place by the Occupational Safety and Health Administration (OSHA) and the American National Standard Institute (ANSI) concerning lasers are recommended to be followed when using LLLT to avoid non-target tissue injury.

## Data Availability

The data supporting the findings of this study are available from the corresponding author upon request.

## Declarations

### Animal welfare

This research was following the animal welfare guidelines.
